
JOURNAL INFORMATION
==============================
NLM Title Abbreviation: ScientificWorldJournal
Journal ID: ScientificWorldJournal
NLM Journal ID: 101131163
Journal ID: TSWJ

The Scientific World Journal

ISSN: 2356-6140
EISSN: 1537-744X
Publisher: Wiley

ARTICLE INFORMATION
==============================
PMCID: PMC6084511
PMID: 30147554
DOI: 10.1100/tsw.2001.134
Article ID: 502945
Article version: 1
Subjects: Short Report

Molecular Regulation of Compromised B Lymphopoeisis in Aged Mice

Blomberg Bonnie B. *
Nguyen Diep
Sherwood Erin M.
Kamm Karen
Perez Marta
Riley Richard L.
Department of Microbiology and Immunology, University of Miami School of Medicine, P.O. Box 016960 (R-138), Miami, FL 33101, USA
*Bonnie B. Blomberg: bblomber@med.miami.edu
Publication date: 2001
Electronic publication date: 2001 Nov 20
Volume: 1
First page: 78
Last page: 78
Copyright: Copyright © 2001 Bonnie B. Blomberg et al.
Copyright year: 2001
License: This is an open access article distributed under the Creative Commons Attribution License, which permits unrestricted use, distribution, and reproduction in any medium, provided the original work is properly cited.
License URL: https://creativecommons.org/licenses/by/3.0/

Funding: National Institutes of Health AG14892 National Institutes of Health AG15474

==============================
Miami *Nature Biotechnology* Short Reports
*TheScientificWorld* (2001) 1(S3), 78SR
ISSN 1532-2246; DOI 10.1100/TSW.2001.134

### MOLECULAR REGULATION OF COMPROMISED B LYMPHOPOEISIS IN AGED MICE

Bonnie B. Blomberg*, Diep Nguyen, Erin M. Sherwood, Karen Kamm, Marta Perez and Richard L. Riley

Department of Microbiology and Immunology, University of Miami School of Medicine, P.O. Box 016960 (R-138), Miami, FL 33101, USA
* bblomber@med.miami.edu

**INTRODUCTION**. The antibody (immunoglobulin, Ig) quality declines with age in both mice and humans. Our laboratory previously determined that pre-B cell numbers as well as the surrogate light (SL) chain, composed of the VpreB and lambda 5 (5) peptides, are reduced in aged mice (1-3). The SL is part of the pre-B cell receptor, critical for Ig variable heavy (VH) chain selection, cellular proliferation, and survival at the pre-B cell stage. We have also established that a human lambda 5 (hu 5) transgene is regulated in a stage- and lineage-specific fashion, similar to that of mouse lambda 5 (m 5) (4).

**METHODS**. Bone marrow cells from 129 mice with the hu 5 transgene of different ages (young, 4 mos to old, 18, 24 and 24+ mos) were analyzed for expression of both m 5 and hu 5. After lysing red blood cells, cells were stained for fluorescent flow cytometry analysis, first on the membrane with CD43-PE and B220-CY to distinguish B lineage subsets, and then for cytoplasmic m 5 and hu 5 after cell fixation and permeabilization with 0.25% paraformaldehyde and 0.2% Tween 20. Reverse transcriptase-polymerase chain reaction (RT-PCR) was also done on total RNA from the same population of cells. Dilutions of the RT were made before PCR, and gel electrophoresis, Southern blotting, hybridization to m 5 and hu 5 probes and quantitation by phosphoimager was performed.

**RESULTS/DISCUSSION**. Here we report that hu 5, as well as m 5, is down regulated in the $CD43^+$ $B220^+$ pro-B/early pre-B cells in aged mice (greater than 18 mos) at the protein level, as measured by flow analysis, and as well, transcriptionally, by RT-PCR. The decrease in m 5/hu 5 coincide with the decreased number of pre-B cells in these aged mice.

These mice provide a model to study the regulation of hu 5 during aging. We have also analyzed the VH repertoire in pre-B cells sorted from aged and young mouse bone marrow in BALB/c mice and show that there are differences in the VH repertoire in aged mice. Therefore, the altered VH Ig changes seen during aging may be in part accounted for by differential VH selection at the pre-B cell stage in the bone marrow, in both mice and humans. Transcription factors E47/E12, shown to regulate 5/ VpreB have also been shown to be downregulated during aging in mature B cells in the periphery (3) and these results are currently being confirmed and extended by gel shift analysis.

**ACKNOWLEDGEMENTS**. This work was supported by the NIH (AG14892 to BBB and AG15474 to RLR).

**REFERENCES.**

1. Riley, R.L., Kruger, M.G., and Elia, J. (1991) Clin. Immunol. Immunopathol. 59, 301
2. Sherwood, E.M., Blomberg, B.B., Xu, W., Warner, C.A., and Riley, R.L. (1998) J. Immunol. 161, 4472
3. Sherwood, E.M., Xu, W., King, A.M., Blomberg, B.B., and Riley, R.L. (2000) Mech. Ageing Dev. 118, 45
4. Donohoe, M.E., Beck-Engeser, G., Lonberg, N., Karasuyama, H., Riley, R.L., Jack, H.-M., and Blomberg, B.B. (2000) J. Immunol. 164, 5269
